# Microalgae: A Promising Source of Bioactive Phycobiliproteins

**DOI:** 10.3390/md21080440

**Published:** 2023-08-04

**Authors:** Latifa Tounsi, Hajer Ben Hlima, Faiez Hentati, Ons Hentati, Hana Derbel, Philippe Michaud, Slim Abdelkafi

**Affiliations:** 1 Enzymatic Engineering and Microbiology Laboratory, Algae Biotechnology Team, Biological Engineering Department, National School of Engineers of Sfax, University of Sfax, Sfax 3038, Tunisia; latifa.tounsi@enis.tn (L.T.); hajer_benhlima@yahoo.fr (H.B.H.); ons.hentati@enis.tn (O.H.); hanaderbel12@gmail.com (H.D.); slim.abdelkafi@enis.tn (S.A.); 2 Université Clermont Auvergne, Clermont Auvergne INP, CNRS, Institut Pascal, F-63000 Clermont-Ferrand, France; 3 INRAE, Animal Research Unit and Functionalities of Animal Products (UR AFPA), University of Lorraine, USC 340, F-54000 Nancy, France; faizhentati@gmail.com

**Keywords:** phycobiliproteins, microalgae, culture, biological activities, biotechnological applications

## Abstract

Phycobiliproteins are photosynthetic light-harvesting pigments isolated from microalgae with fluorescent, colorimetric and biological properties, making them a potential commodity in the pharmaceutical, cosmetic and food industries. Hence, improving their metabolic yield is of great interest. In this regard, the present review aimed, first, to provide a detailed and thorough overview of the optimization of culture media elements, as well as various physical parameters, to improve the large-scale manufacturing of such bioactive molecules. The second section of the review offers systematic, deep and detailed data about the current main features of phycobiliproteins. In the ultimate section, the health and nutritional claims related to these bioactive pigments, explaining their noticeable potential for biotechnological uses in various fields, are examined.

## 1. Introduction

Microalgae encompass a wide group of photosynthetic microorganisms, which consist of both prokaryotic cyanobacteria and eukaryotic microalgae. These organisms utilize the process of photosynthesis to convert light energy and carbon dioxide (CO_2_) into chemical energy [1]. Microalgae have garnered considerable interest due to their high growth rate and their ability to produce numerous biomolecules including proteins, lipids, carbohydrates and pigments. Microalgae are a source of industrially important products like feed, food, dyes, nutraceuticals, cosmetics, pharmaceuticals, etc. Artificially or chemically synthesized dyes have been broadly utilized in the pharmaceutical, cosmetic, textile and nutraceutical fields. However, these chemically synthesized dyes constitute serious health risks [2]. Thus, to avoid the harmful impacts of artificial dyes, the use of natural pigments obtained from organisms like microalgae is greatly encouraged.

Pigments refer to intricate molecules or macromolecules capable of changing the color of light that they transmit or reflect through selective absorption of specific wavelengths. In microalgae, natural pigments serve a crucial function in various activities reliant on pigmentation, including mechanisms related to photosynthesis [3,4]. They have substantial potentials as biologically active compounds, food colorings and nutraceutical ingredients with antioxidant, anticancer, immunomodulatory, antiangiogenic, antidiabetic, and anti-inflammatory properties. Microalgae contain three categories of pigments: (*i*) chlorophylls, (*ii*) carotenoids and (*iii*) phycobiliproteins (PBPs). Chlorophylls, which are the primary pigments responsible for photosynthesis process, can be classified into four types (a, b, c and d) and have distinct molecular structures. These chlorophylls are fat-soluble and give a greenish color to the microalgae. Carotenoids and PBPs, on the other hand, are accessory pigments. Chlorophyll b and diverse kinds of chlorophyll c are present in green algae (Chlorophyceae) and brown algae (Phaeophyceae), respectively [5], whereas chlorophyll d, as well as other main accessory pigments, such as R-phycocyanin (R-PC), allophycocyanin (APC) and carotens (α/β), are found in red algae (Rhodophyta) [6]. The main microalgal carotenoids include carotenes, fucoxanthin, astaxanthin, lycopene, neoxanthin, zeaxanthin, lutein and violaxanthin. PBPs, which are abundant accessory pigments involved in photosynthesis, consist of phycocyanin, which has an intermediate energy level; phycoerythrin (PE) or phycoerythrocyanin (PEC), which has high energy levels; and allophycocyanin, which has a low energy level [7,8]. PBPs exhibit several distinct qualities, including (*i*) a high extinction molar coefficient, (*ii*) a significant Stokes shift, (*iii*) minimal suppression of fluorescence, (*iv*) a high fluorescence quantum efficiency and (*v*) wide-ranging absorption across the visible light spectrum [9]. Due to these characteristics, PBPs have emerged as promising fluorescent labeling agents suitable for applications in fluorescence microscopy, flow cytometry, immuno-histochemistry, fluorescence immunoassay and various other biomedical studies [10,11,12]. Red PBPs, namely R-PE and B-PE, can be obtained through isolation from red microalgae such as *Rhodella* spp., *Bangia* spp. and *Porphyridium* spp. On the other hand, blue PBPs, known as APC, can be extracted from *Spirulina* spp. in significant quantities. Biosciences, Algapharma Biotech and QuantaPhy are renowned as pioneering manufacturers and suppliers of PBPs utilized as fluorescent labeling agents. Nowadays, the market of drug development is more interested in PBPs for pharmaceutical applications. The cost of PBPs varies based on factors such as their purity and intended application, with prices ranging from USD 130 to USD 15,000 per gram. However, when PBPs are used for food-related purposes, the requirement for high purity is often less stringent, allowing for the possibility of reducing costs. When used for food applications, the purity is not really a constraint and the cost can be easily decreased. When employed in therapeutic and scientific applications, the purity needs to be higher and the price can be a hundred times greater [13]. The PBP total market value was more than USD 60 million in 2013 [14]. According to a Future Market Insights report, the PBP global market size reached USD 112.3 million in 2018, and it is projected to grow twofold by 2028 [15]. According to the same report, Western Europe stands out as the largest consumer of this product, accounting for approximately 33% of the consumption. Moreover, the food industry utilizes around 80% of the produced phycocyanins. 

The present review aims to provide an extensive overview of (*i*) culture key parameters, which impact the production of microalgal PBPs; (*ii*) the most relevant features of PBPs; as well as (*iii*) their associated applications, with a specific focus on their biological activities.

## 2. Impact of Culture Conditions on PBP Production by Microalgae

A variety of biotic and abiotic conditions impact both growth and metabolites production by microalgae. In terms of abiotic factors, temperature, light, nutrient’s concentration, pH, salinity and medium composition can change the metabolism pathways of the organism. Microalgae evolved as organisms capable of extreme adaptation to diverse environmental conditions. These adaptations are commonly associated with the development of a defense mechanism that leads to the production of various valuable compounds and natural products. Such metabolites are influenced by the stress factors that the organism experiences, and the effective production of a specific metabolite requires the careful consideration and optimization of various factors related to the microorganism’s culture conditions [16]. Fixing the key elements for biomass production will contribute to ensuring ideal cultivation conditions, leading to significant advancements in the economic utilization of biomass resources.

As photoautotrophic microorganisms, light is the main energy source for microalgal growth, allowing important metabolic processes to be carried out (e.g., photosynthesis). The primary metabolic processes, including the density change in biomass and the accumulation of compounds, take place specifically during photosynthetic activity and other light-regulated pathways, with varying wavelengths of light having been demonstrated the ability to increase the production of specific compounds [17]. Therefore, light optimization in order to enhance the production of PBPs can be mainly associated with light intensity, quality (spectra composition) or the period of exposition. Keithellakpam and colleagues [18] found that *Nostoc muscorum* produced more PCs when exposed to red light and more PEs when exposed to green light. This might be easily explained by the need for cyanobacteria to absorb a particular range of light in order to ensure the efficacy of photosynthetic processes. The same effect was also seen in the case of *Pseudanabaena* sp. [19]. However, the acclimation to various light qualities is not horizontal and differs depending on the species. It has been described that some *Anabaena* species enhance total PBP productivity when exposed to blue light [20,21]. Similar effects have been documented in *Synechococcus* sp. [22] and *Nostoc sphaeroides* [23]. In their study, Baer et al. [24] studied the impact of 37 different light quality conditions on the productivity of cell dry weight and PBPs of *Porphyridium purpureum*. They found that a composition of α_red_:d_green_:e_blue_ in a ratio of 40:40:20 resulted in the highest PE and biomass productivity, with respective values of 16.93 and 311.6 mg/L/day.

Coward et al. [25] investigated the impact of specific narrow light-emitting diode wavelengths (red, blue and green) and a combination of LED wavelengths on biomass composition produced by *P. purpureum*. The findings demonstrated that green light was crucial for the growth and accumulation of PBPs, which can be explained by the fact that PBPs can absorb green wavelengths when chlorophyll is poorly absorbed. Additionally, light intensity is one of the most crucial factors to be optimized in order to obtain a sustainable microalgal culture. Concerning the target production of PBPs, it has been broadly reported that a preference for low and medium light intensities was found for the biosynthesis of these pigments [20,23,26,27]. The role of these compounds in the process of photosynthesis accounts for this phenomenon. PBPs are synthesized to expand the range of light absorption. In low-intensity conditions, the microorganism will require alternative means to obtain light and energy in order to grow. It has been reported by Mihova et al. [28] that biomass production by *Rhodella reticulata* increased in response to the increase in light intensity, whereas PBP content decreased under the same conditions. Jahn et al. [29] studied the changes in the PBP content of *P. cruentum* and *Porphyridium aerugineum* at light intensities of 220 and 3200 μW/cm^2^ and found that the cells, which were grown under low light intensity accumulated up to three times more PBPs than microalgal cells cultured at high-light-intensity conditions. The same effect was also seen for *Porphyridium marinum* [11]. Also, high light levels can result in photoinhibition, as seen with *S. platensis* [30], which was unable to grow in a light intensity above 4000 LUX. When the energy level is higher than the capacity supported by the microalga, the charge inside the cell is excessive and enhances the ROS formation, creating a toxic environment and, consequently, causing cell death [31]. Some microalgal species and strains, however, are less light-sensitive and can grow under high light intensity, and this high level of energy can promote the growth, as well as metabolism, of these microalgae. 

The positive impact of high intensity levels was observed by Chaneva et al. [32] and Derbel et al. [33], where *Arthronema africanum* and *Rhodomonas* sp. accumulated more PBPs under light intensities, higher than 150 μmol photons/m^2^/s. A few other investigations showed the same preference of other microalgae for high light intensities [34,35]. Additionally, the duration of illumination and the intensity of light received by the organism have an effect on photosynthetic activity and, consequently, on microalgal metabolism. There is indeed a relation between the amount of light the microorganism receives each day and the illumination duration. In fact, a relation exists between the light intensity that the organism receives per day and the illumination duration. It appears that microalgae can be exposed to a minimal photon rate per day to optimally grow during a long photoperiod [36]. In regard to PBP, it has been consistently observed that a period of darkness is necessary. It was proposed that a photoperiod of 16h of light and 8h of darkness was the optimal culture condition for the accumulation of PBPs [20,35,37].

Besides light, temperature stress also strongly affects the biochemical composition of microalgae. Thus, the ideal conditions for growth or metabolite production depends on the tolerance and adaptation of each microalgal strain [20]. The effects of temperature on the accumulation of PBPs have not been deeply investigated in various species. The accumulation of PBPs by microalgal species such as *A. africanum* [32], *Nostoc* sp. [37] and *S. platensis* [34] seems to increase under temperatures equal to or greater than 30 °C. Sakamoto and Bryant [38] noted that *Synechococcus* sp. PCC 7002 displayed greater sensitivity and a preference for low temperatures, specifically 22 °C.

Moreover, pH plays an important role in the growth of microalgae as it influences their metabolic, as well as biochemical, activities. The effects of pH fluctuations on numerous cellular and extracellular processes have been extensively documented by numerous researchers [16,18,39]. These processes encompass solubility, substance transport across the cell membrane, bioavailability of nutrient, intracellular and extracellular enzyme activity, photosynthetic electron transport and cytoplasmic osmotic potential. The latter processes are the factors that affect the metabolic reactions of PBP biosynthesis the most [40,41]. The pH range for PBP biosynthesis is between 6 and 10, but the most appropriate pH range is the alkaline region. For instance, bleu microalgae have an effective pH range between 7.1 and 7.5 [37]. In general, pH influences the production of PBP much more than the growth of microalgae. In this context, Johnson et al. [37] have observed that *Nostoc* sp. did not grow below a pH of 3 or beyond a pH of 12 and that the most suitable pH for PBP biosynthesis is 8. Extreme pHs can denaturize proteins [18]. The optimal pH value is 7.0 for *Rhodomonas* sp. [33]; 8.0 for *Nodularia sphaerocarpa* [42], *Nostoc muscorum* [18], *Gloeocapsa* sp., *Synechocystis* sp., *Anabaena* sp. and *Lyngbya* sp. [35]; 9.0 for *Spirulina* sp. [43], *Nostoc* sp. [44] and *S. platensis* [45,46]; 9.5 for *Anabaena fertilissima* [21]; and 10.0 for *Synechocystis* sp. [47]. 

Some growth media such as Artificial Sea Water [48], BG11 [49] and Zarrouk synthetic medium [50] have been described to be very suitable and effective for the growth of microalgae, biomass production and PBP accumulation. Thus, the selection of an appropriate culture medium is a very important factor that must be taken into account depending on the microalgae species. In fact, it is crucial to provide appropriate nitrogen and carbon sources as they both play significant roles in the metabolism of biomass production and PBP accumulation. Borsari et al. [51] described the enhancement of microalgal production in mixotrophy for *Nostoc* sp., where the addition of sugar boosted the production of PBPs by a factor of 12. The utilization of carbon sources like glucose, fructose, sucrose and glycerol for industrial-scale production of microalgae under mixo- or heterotrophy may be economically possible for the production of fine chemicals, high-valued bioactive compounds and pharmaceuticals [52]. Venugopal et al. [53] showed that the addition of sucrose and glucose under low light intensities enhanced the content of PBPs in *Anabaena azollae*. However, when the light intensity was greater, the addition of sugars was ineffective. Kovač et al. [52] compared the impact of glycerol and glucose on the growth of six microalgae and revealed that the species behaved differently from these additions in their culture media. 

Besides sugars and glycerol, the carbon supply can originate from the injection of CO_2_. Zeng et al. [54] reported that the introduction of CO_2_ into the culture medium of *Spirulina platensis* resulted in enhanced growth and increased production of PBPs. This phenomenon can be attributed to the organism’s improved ability to regulate its nutrition and metabolism. In addition, the presence of CO_2_ stimulates carboxylase activity of Rubisco to fix carbon and to generate biomass. The absence of a carbon source can help in PBP accumulation. It was observed by Sharma [55] that carbon shortage resulted in an increased accumulation of PBPs. This is due to the fact that these pigments also serve as a defense mechanism for the microorganism, protecting the photosystem membrane against damage caused by light when carbon assimilation is restricted in stressful conditions. The source of nitrogen in the culture medium can also affect metabolite production as it changes the assimilation mechanism of nutrients inside the cell. Only a few investigations have evaluated the production of PBPs using various nitrogen sources. Khazi et al. [56] investigated PBP production and biomass production by three microalgae when potassium or sodium nitrates and ammonium chloride were used as nitrogen sources. The findings revealed that, for *Arthrospira platensis*, the greatest PBP content was reached using NaNO_3_, whereas both strains *Pseudoscillatoria* sp. and *Phormidium* sp. accumulated better PBP contents with ammonium chloride. Related to the nitrogen source, the supplementation harmed the accumulation of PBPs in some strains [20,21,57]. Hemlata and Fatma [20] showed that *Anabaena* sp. accumulated more PC when no nitrogen source was supplemented to the culture media. Cyanobacteria like *Spirulina* spp. and *Calothrix* sp. revealed a decrease in PBP production when the sodium nitrate concentration increased in their culture media [16,26,58]. On the other hand, for some species including *Phormidium ceylanicum* [59], *Limnothrix* sp. [60], *Oscillatoria* sp. [61], *P. marinum* [11], *Rhodomonas* sp. [62] and *Euhalothece* sp. [63] a direct correlation between sodium nitrate concentration in the culture medium and PBP accumulation was observed. 

During growth media optimization, various variables have to be taken into account to control the growth and the accumulation of a large amount of PBP in microalgae, among them carbon and nitrogen sources, without forgetting about other culture media compositions including salinity, vitamins and trace metals being essential. Salinity is a fundamental factor for growth, as well as for metabolite production, by microalgae. It can change within regions, and thus, every species has its own adaptation. Only few studies focusing on the impact of salinity on the production of PBPs have been realized. Hemlata and Fatma [20] have revealed that PBP production by *Anabaena* NCCU-9 increased under low concentrations of NaCl (0.01 M), whereas an increase in salinity (0.25 M) caused a reduction in the production of these pigments. Lemus et al. [60] have also reported the same negative impact of the increase in salinity for *Limnothrix* sp. Additionally, the rapid intake of sodium ions may cause the detachment of phycobilisomes from the thylakoid membranes, which may result in a decrease in the rate of photosynthetic activity and, consequently, an absorption of other mineral nutrients [20,64]. A significant impact of the salinity on the growth, as well as phycobilins accumulation, has been reported for *Phormidium* sp., where the greatest amount of PBPs was in seen with average sea salinity [65]. Another investigation studied the role of various concentrations of salt on PBP content in *Synechocystis* sp. PCC 7338, and it was revealed that the treatment with NaCl (1.2 M) produced higher contents of both APC and PE [66]. 

## 3. Phycobiliproteins from Microalgae

**Figure 1:**
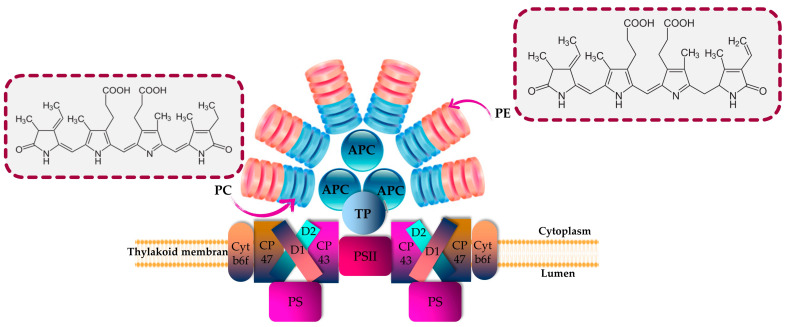
Structural and energetic funnel models of phycobilisome. APC is allophycocyanin; cyt is cytochrome; D1 and D2 are proteins related to chlorophyll; PC is phycocyanin; PE is phycoerythrin; and TP is terminal pigment.

PBPs play a significant role as accessory pigment complexes, primarily responsible for capturing additional light in the visible spectrum for enhanced light harvesting. They are predominantly present in various groups of cyanobacteria and eukaryotic algae, including Rhodophytes, Glaucocystophytes and Cryptomonads (the phylum Cryptophyta) [16,33]. In addition, these fluorescent proteins offer protection to microalgal cells against photolysis caused by exposure to high-intensity light [67,68]. PBPs are frequently found in *Porphyra*, *Asparagus schoberioides*, *Polysiphonia urceolata*, *Spirulina platensis*, *Porphyridium* and *Microcystis* [9,69,70]. In Cyanobacteria and Rhodophyta, PBPs are arranged into phycobilisomes, as depicted in Figure 1, whereas in Cryptomonads, the phycobiliprotein antenna is typically separate and located within the thylakoid lumen [33].

PBPs are characterized by being biodegradable; hydrophilic; nontoxic; and brilliantly colored biliproteins that consist of phycobilins, which are tetrapyrrole chromophores with open-chain structures [16,71]. According to their characteristics related to light absorption, PBPs are commonly classified into four distinct categories: phycoerythrins (pink-purple; λ_max_ = 540–570 nm), phycoerythrocyanins (PECs; orange; λ_max_ = 560–600 nm), phycocyanins (blue; λ_max_ = 610–620 nm), and allophycocyanins (blue-green; λ_max_ = 650–655 nm) [11,33] (Figure 1). PEs are categorized into three primary classes based on their origin and absorption spectra: R-phycoerythrin (R-PE) (peaks of absorbance at 499 and 565 nm and a shoulder at 545 nm), B-phycoerythrin (B-PE) (peaks of absorbance at 545 and 565 nm with a shoulder at 499 nm) and C-phycoerythrin (C-PE) (peak of absorbance at 565). Accordingly, the prefixes “B” and “R” were assigned to PBPs based on the taxonomic origin of the organism from which they were extracted. Specifically, the designation “R-PE” was given to phycoerythrins derived from Rhodophyta, while “B-PE” was used for those obtained from the Bangiales order of Rhodophyta [72].

PBPs with different energy levels exhibit significant overlap in their absorption and fluorescence emission spectra [73]. Within the phycobilisome, energy transfer occurs sequentially from phycoerythrin, phycocyanin and allophycocyanin towards the photosynthetic reaction center. Indeed, the light energy absorbed by phycobilins is transmitted initially within the subunits, then among different PBPs and ultimately to the reaction center located in the thylakoid membrane. The energy transfer efficiency exceeds 95% [74,75]. As a mediator of energy transfer through the photosystems, APC absorbs the energy from the preceding PBPs and passes the energy to photosystem I (PSI). PBPs offer several advantages, such as their excellent water solubility, high fluorescence quantum yield, non-toxic nature, significant Stokes shift and resistance to rapid fluorescence quenching [73]. Additionally, after cross-linking with other proteins, the spectral characteristics and quantum yield are generally maintained. PBPs consist of either two or three subunits. PE is a monomer (αβ) composed of two subunits (α, β) combined via electrostatic attraction, and three monomers are then joined to form a stable aggregation. In the middle of PBP, there is a trimer (αβ)_3_ or two trimers that are recombined to create a more stable hollow hexamer (αβ)_6_. PEs comprise not only α and β subunits but also a γ subunit. PE is often found in the form of hexamer (αβ)_6_γ [76,77]. PBPs have a molecular weight (M_w_) between 220 and 300 kDa. The α subunit size is approximately between 13 and 20 kDa; the β subunit is larger, with an M_w_ between 14 and 24 kDa; and the γ subunit in PE measures about 30–34 kDa, making it more stable [73]. Cyanobacteria, also known as blue-green microalgae, constitute the main source of PCs, which are a blue pigment–protein complex [78]. *Spirulina* is one of the most important sources of C-phycocyanin (C-PC). Also, *Galdieria sulphuraria* is a promising candidate for heterotrophic production of phycocyanin [79]. PCs have found a wide range of applications in the nutritional, cosmetical and pharmaceutical fields, where they are widely employed as natural dyes. They are highly suitable for use as a non-toxic fluorescent reagent for immunoassays and serve as fluorescent probes for analyzing molecules and cells [78]. Furthermore, their rich protein content positions them as promising candidates for applications in the food industry [80]. APC is located in the core of the phycobilisome, present in all phycobiliprotein-containing organisms [81]. The total content of APCs is relatively less compared with that of PCs and PEs in red algae, cyanophytes and glaucophytes. They exist as heteromonomers (αβ), with an M_w_ ranging from 17 to 20 KDa [82]. Within microalgal cells, biosynthesis of PBPs is carried out through the transcriptional, translational and posttranslational pathways, which lead to the biosynthesis of amino acids, proteins and phycobilins. Finally, in the post-translational stage, the process involves the attachment of phycobilins to apoproteins, resulting in the formation of PBPs [83]. Phycobilins are produced via the biosynthesis process starting with heme. Heme oxygenase plays a role in converting heme into biliverdin. Subsequently, other enzymes facilitate the conversion of biliverdin into the phycobilins [84]. The ultimate stage in the biosynthesis of PBPs involves the covalent attachment of phycobilins to particular cysteine residues through thioether bonds facilitated by bilin lyases.

Given that the pigment content is often a distinguishing feature of each microalga [85], it becomes evident that a unique extraction method should be employed for each investigation. Over the past few decades, a multitude of protocols have been developed, alongside comparisons of different methods, to extract and measure pigments. PBP recovery from algal biomass is carried out via numerous stages, which can be summarized into four principal steps, namely, (*i*) cell disruption, (*ii*) extraction, (*iii*) purification and (*iv*) characterization of PBPs as final products. Each of these phases uses a different set of specific methodologies and techniques. The extraction of PBPs could be difficult due to the cell wall resistance, as well as the small size of microalgae. To initiate the extraction of PBPs, the initial stage involves choosing an appropriate technique that effectively releases PBPs from microalgal cells while minimizing any significant alterations to their structures and functions. Typically, there is a positive correlation between the proportion of broken microalgal cells and the yield of PBPs. In other words, as the percentage of broken cells increases, the yield of PBPs also tends to increase. Nevertheless, employing violent cell disruption methods can potentially adversely affect the structural integrity and functionality of PBPs. It has been reported by Tran et al. [86] that high-pressure treatment could provoke B-PE denaturation. Mechanical (e.g., grinding, bead milling, ultrasonication and high-pressure homogenization) and/or non-mechanical techniques (e.g., lysozyme treatment, repeated freezing and thawing, and osmotic shock) are the main used methods for cell disruption (Table 1) [87,88]. The selection of methodology must take into consideration factors including the microorganism’s composition, as well as the resistance and stability of its cell wall [89]. A comparison of different extraction methods for extracting C-PC from *S. platensis* demonstrated that the efficiency of extraction using lysozyme treatment, repeated freezing–thawing, and bacterial (*Klebsiella pneumoniae*) treatment was comparable; however, sonication and glass bead grinding proved to be ineffective [90]. In another study, researchers examined various extraction techniques for obtaining PBPs from the marine microalga *Porphyridium cruentum* [91]. They discovered that buffer extraction from lyophilized microalga outperformed other tested extraction techniques like repeated freezing–thawing cycles and sonication in terms of effectiveness. In laboratory settings, it was common practice to enhance the extraction efficiency by employing a combination of different approaches. The extraction of PBPs from microalgal cells can be carried out using either wet or dry biomass [92]. It has been suggested that extracting PBPs from wet microalgal biomass is preferable since it helps to prevent pigment loss that can occur during several drying processes [92,93,94,95,96]. Additionally, the cost of extracting from wet biomass is lower compared with extracting from dry biomass, primarily because of the additional expenses associated with drying procedures [97]. For instance, extraction from dry algal biomass might cause a loss in PC of approximately 50% and, finally, can cause variations in the spectra in comparison with PC from wet biomass [95,96]. 

After the cell disruption stage, the supernatant has to be collected for further extraction and purification. Four sets of equations previously described by Kumar et al. [109], Barufi et al. [110], Munier et al. [111] and Baghel et al. [112] are usually employed to quantify PBPs. In a crude extract, the PBP concentration is relatively low. Therefore, additional work must be carried out to increase the PBPs’ purity. The purification procedure frequently involves multiple stages and techniques including precipitation, two-phase aqueous extraction, ultrafiltration, gel filtration and chromatography (Table 2).

**Figure 2:**
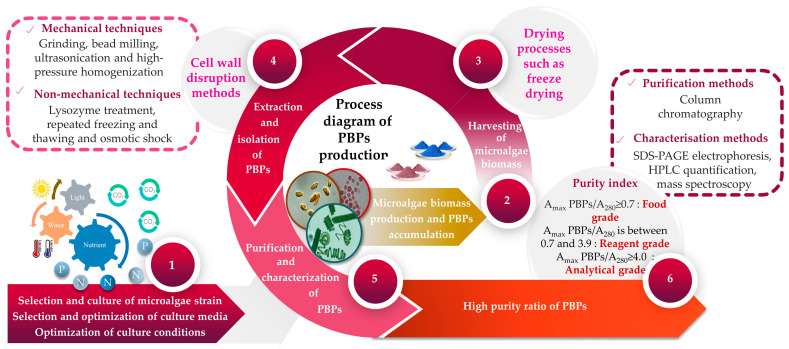
A flowchart illustrating the upstream and downstream processes involved in the production of microalgae-based PBPs.

PBPs should be purified from a desalted extract by using ion exchange and adsorption chromatography [122]. Finally, the PBP is characterized using SDS-PAGE and absorption spectra (at wavelengths 562, 620 and 652 nm for PE, PC and APC, respectively) [122]. The purity of PBPs determines their potential applications. PBPs are categorized as food grade when the ratio of maximum optical density of PBP (DO_max_ PBP) to optical density at 280 nm (DO_280_) is equal to or greater than 0.7, as reagent grade when the DO_max_ PBP/DO_280_ ratio falls between 0.7 and 3.9, and as analytical grade when the DO_max_ PBP/DO280 ratio is equal to or greater than 4.0 [123]. According to Gaignard et al. [36], the purification flowchart of B-PE (and R-PC) from the microalgal biomass includes the following steps: (*i*) cell disruption followed by a centrifugation; (*ii*) filtration of the supernatant before precipitation with ammonium sulfate (65%, 12 h); (*iii*) centrifugation (4500 g, 10 min) to obtain a pellet, which will be resolubilized in specific buffers (acetic acid-sodium acetate, pH 5.5) and then dialyzed; (*iv*) other centrifugations to ultimately obtain a supernatant injectable in ion exchange chromatography (DEAE-cellulose columns); and (*v*) the collection of PE and PC fractions (Figure 2).

In order to reduce the number of unitary operations required to purify B-PE, Benavides and Rito-Palomares [124] have developed two-phase processes to avoid chromatography and (NH_4_)_2_SO_4_ precipitation. The procedure includes cell lysis using sonication and B-PE recovery via aqueous two-phase partition using polyethylene glycol (PEG). Exopolysaccharides, as well as cell-bound polysaccharides (PS), are the principal drawback limiting these B-PE purifications since they change the solubility of this pigment and the behavior of red microalgae during their lysis [125]. To avoid this problem, only cells in the exponential growth phase are employed to extract and purify B-PE in order to limit the presence of PS [125]. 

## 4. Pharmaceutical Potentials of Phycobiliproteins

**Figure 3:**
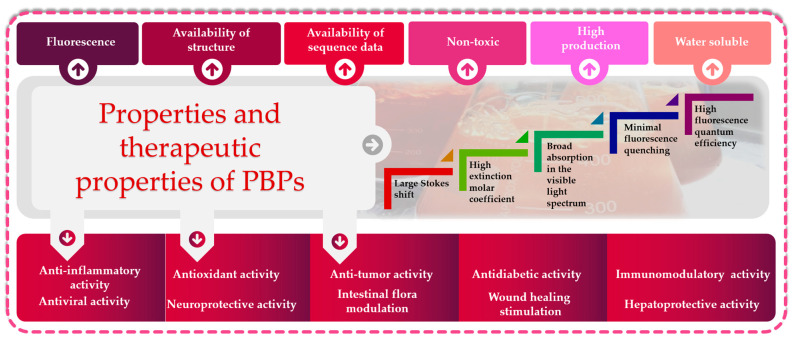
Functional and biological properties of phycobiliproteins: great potential for biotechnological applications.

In the last decade, PBPs have been broadly employed as additives, fluorescent probes and natural colorants. Several investigations have reported the biological activities of PBPs, including antitumor, antioxidant, hepatoprotective and neuroprotective activities, and photosensitizers for tumor treatment can be developed (Figure 3). 

### 4.1. Antioxidant Activity 

The metabolic response to oxidative stress may have an impact on cellular elements, including nucleic acids, proteins and the cell membrane. It can result in a variety of illnesses like diabetes, cardiovascular illnesses, inflammation, cancer, degenerative illnesses, ischemia and anemia [126]. For the prevention and treatment of these illnesses, only a few phytochemicals have already been employed, e.g., caffeic acid, α-tocopherol and zeaxanthin. According to Hirata et al. [127], phycocyanobilin (PCB) has a high potential as an antioxidant as long as its effectiveness is comparable with that of other phytochemicals. The organisms generate antioxidant compounds as part of their defense system to counteract the harmful effects of oxidation. Reactive oxygen species (ROS) accumulation is the primary factor contributing to oxidative stress. The neutralization of ROS is managed through both enzymatic and non-enzymatic antioxidant mechanisms. Compounds like PC can operate as non-enzymatic antioxidants, generally by scavenging the free radicals of ROS, neutralizing their active molecules and reducing the level of oxidation. More specifically, oxidative stress may harm DNA and result in apoptosis or mutagenesis. Both PC and PCB from *S. platensis* are effective at scavenging peroxynitrite and inhibiting DNA damage [128]. Recent studies have revealed that C-PC has antioxidant action in a variety of applications, including the avoidance of oxidative stress in acute kidney damage brought on by HgCl_2_ [129] and the reduction in acute liver oxidative damage from X-rays [130]. Benedetti et al. [131] have shown the strong antioxidant properties of PCB, as well as PC, using the Oxygen Radical Absorbance Capacity technique, which indicates a positive antioxidant profile for certain biological samples. Zhou et al. [132] and Patel et al. [68] demonstrated the capacity of PBPs to scavenge free radicals. PBPs were thought to effectively chelate and reduce the ferrous ion, indicating the combined involvement of the metal-ion-chelating and electron-donating capacities of the PBP-constituting amino acids in expressing antioxidant potential [127]. Huang et al. [133] obtained selenium-containing PCs isolated from selenium-rich *S. platensis* and investigated their antioxidant properties, including their capacity to scavenge superoxide free radicals, hydrogen peroxide and 2,2-diphenyl-1-picryl-hydrazyl free radical (DPPH). The results showed that the antioxidant capability of selenium-PCs varied when exposed to different types of free radicals, and the scavenging potential of selenium-PCs for hydrogen peroxide and superoxide radicals exhibited a positive correlation with the selenium content. According to Gdara et al. [134], PCs decreased liver damage by lowering the activity of oxidative stress-activated enzymes like alkaline phosphatase and liver transaminases. In addition, the PCs described by Praneel [135] were not only considered as great antioxidants but also as anti-inflammatory and immune stimulant agents. Cervantes-Llanos et al. [136] have demonstrated the utility of PCs as neuroprotectors in rodent models with autoimmune encephalomyelitis, where it decreased oxidative stress and immunological response. The antioxidant effects are impacted by many factors like pH, light and denaturing agents. PC generated hydroxyl radicals when exposed to light; however, in the case of darkness, PC scavenged the hydroxyl radicals. An increase in pH above 7.0 or denaturation of PCs by urea or sodium dodecyl sulfate (SDS) led to the loss of the ability to generate hydroxyl radicals and, concurrently, an augmentation in antioxidant activity [132].

### 4.2. Antitumor Activity

**Figure 4:**
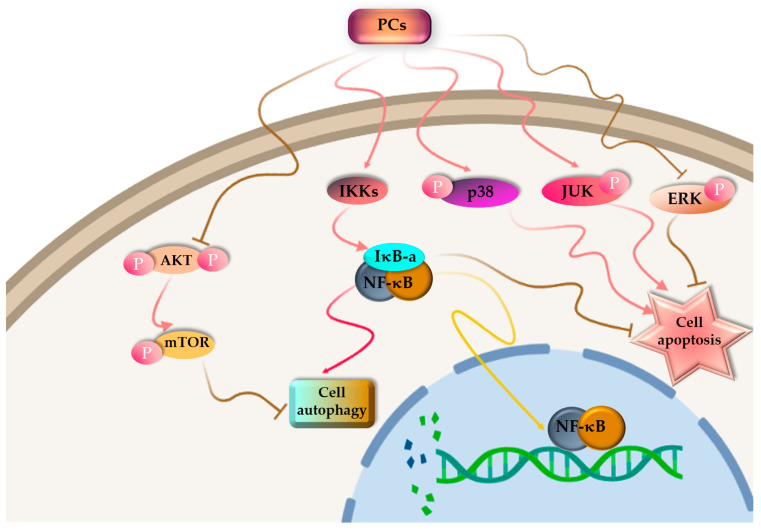
Proposed pathway mechanisms for PC-induced autophagy and apoptosis in cancer cells.

Cancer is one of the leading causes of death and constitutes an enormous social burden worldwide. Surgical resection is currently the preferred option for treatment in early-stage cancer, whereas chemotherapy is essential for treating advanced cancer. At the cellular level, cancer cells are characterized by unending cell division, apoptotic incapacity and invasion of cell growth. As a result, cancer treatment can be achieved through the induction of tumor cell apoptosis and cell cycle arrest, inhibition of tumor cell proliferation and inhibition of the migration of tumor cell. For the past few decades, there has been significant research focused on investigating natural products derived from plants, fungi, algae and cyanobacteria, both in the form of pure compounds and extracts. The outcomes of these studies have been highly encouraging [137,138]. Among PBPs, PCs have been investigated for their antitumor activity (Figure 4).

PC is poisonous to cancer cells, but it has no side effects on normal cells [139,140]. PBPs can influence the cell cycle, resulting in cell cycle arrest. Liu and colleagues [141] documented that C-PC derived from *S. platensis* demonstrated a suppressive impact on the proliferation of K562 cells, which are associated with human chronic myelogenous leukemia-blast crisis. The progression of these cells through the S-phase was halted, leading to their arrest at the G1 phase. More and more evidence has demonstrated that PCs have an effective antitumor impact on different kinds of cancerous cells such as liver cancer [142], leukemia [143], breast cancer [144], lung cancer [145], colon cancer [146] and bone marrow cancer [147], both in Vitro and in Vivo assays. Novel cancer-targeted nano-drug C-PC/CMC-CD59sp nanoparticles were created by Jiang et al. [148] using carboxymethyl chitosan (CMC), C-PCs and the CD59-specific ligand peptide (CD59sp). It was reported that the C-PC/CMC-CD59sp nanoparticles could induce G0/G1 cell cycle arrest and decrease the growth in cervical cancerous cells (SiHa and HeLa), and the proliferation of cancer cells was decreased in a dose-dependent manner.

It has been found by Tan et al. [149] that R-PE induces apoptosis by keeping the SGC-7901 cell cycle at the S phase, which was achieved by reducing the expression of the CDC25A protein, decreasing the CDK2 protein activity and forming the cyclin–CDK complex. According to Hao and colleagues [150], PC produced by *S. platensis* may impact the metabolism of non-small-cell lung cancer cells. Finally, it induces apoptosis of tumor cells and has an impact on the activity of serine/threonine protein kinase I and NF-B signal expression. As a result, PC inhibits the growth of lung cancerous cells, as well as their proliferation ability. Many investigations have demonstrated that using two or more pharmacological combinations to treat the disease can increase the therapeutic efficiency [151]. 

During tumor treatment, different drug combinations can significantly increase the safety and efficiency of a single agent in a tumor therapy regimen. Potentially, PC can boost the effectiveness of cancer-fighting drugs already on the market. It is reported that PC can kill human tumor cells when combined with a variety of drugs and radiation. Gantar and co-workers [152] treated a prostate cancer cell line, LNCaP, with the anticancer drug topotecan (typical dose of 10%) and C-PC isolated from *Limnothrix* sp. 37-2-1. The result of the treatment was better than that using a single C-PC or a topotecan treatment. Similarly, the combination of these two compounds reduced free ROS levels and increased the activities of a large number of caspase-3 and -9. It also induced tumor cell apoptosis and decreased the harsh side effects of topotecan in patients. Also, Saini et al. [153] showed that the combined effects of piroxicam and PC administration was more potent than the single-use drugs when treating 1,2 dimethylhyadrazine-induced rat colon carcinogenesis, and the impact was 70% greater than that of single-use drugs. Cyclooxygenase 2 expression, DNA fragmentation and the prostaglandin E2 levels were remarkably decreased and the size and number of tumors were also reduced. In that regard, it has been suggested that PC can potentially enhance the effectiveness of the currently available antitumor drugs. Furthermore, the PC light-absorbing property has the potential to be employed as a photosensitizer in drug delivery applications, as demonstrated in a study conducted by Wan et al. [154]. The investigation revealed that the combination of zinc phthalocyanine with PC produced significant outcomes in terms of photodynamic activity and targeted effectiveness against cancerous cells.

### 4.3. Anti-Inflammatory Activity

Inflammation is a dynamic immune system response feedback to external or internal damage and infection [155]. Generally, the inflammatory process is present in everyday human life. This process can result in moderate symptoms like muscle pain or severe ones like acute lung injury, which is marked by destruction of the epithelial and endothelial cells in the lungs and can be fatal for patients in intensive care units [156]. The primary source of pro-inflammatory signals in cases of inflammatory disorders is macrophage induction. The anti-inflammatory capacity of PBPs is associated mainly with the action of these molecules in numerous mechanisms of action, in enzyme expression and activation, and in modulation of macrophages function, inhibiting the pro-inflammatory signals. C-PC isolated from *Arthospira maxima* presented an anti-inflammatory impact in a mouse arthritic model induced by azymosan [81]. Recently, Lu and co-workers [157] employed radiation-induced severe intestinal injury to mice in order to investigate the PC mechanism in the prevention of intestinal inflammation. The findings showed a considerable improvement in the radioradiated intestinal damage brought on by radiation with 12 Gy X-ray following PC therapy. Meanwhile, PC may also control the balance between the harmful and beneficial bacteria in the gut microbiota, thus lowering the levels of lipopolysaccharide, inhibiting the TLR4-Myd88-NF-κB pathway and protecting against intestinal damage brought on by excessive radiation. Additionally, mice colitis induced by dextran sodium sulfate was studied by Zhu et al. [158], and the findings revealed that a daily supplementation of 150 mg/kg of selenium-enriched PC (Se-PC) from *S. platensis* could successfully inhibit dextran sodium sulfate-induced colitis and reduce bloody diarrhea and weight loss in the affected mice. This activity of Se-PC is thought to be able to reduce the release of inflammatory factors in the gut, to inhibit the expression of NF-κB and therefore to activate macrophages. Xia et al. [159], in 2016, fed female KM mice with 30% ethanol to elicit symptoms of alcoholic liver disease and evaluated the preventive impact of PC derived from *S. platensis*. They revealed that PC decreased the blood levels of aspartate aminotransferase (AST) and alanine aminotransferase (ALT). The serum AST and ALT levels of the model group were increased by 96.72% and 113.6%, respectively, from those of the control. Compared with alcoholic liver mice, different doses of PC were able to lower the levels of AST and ALT. Among them, the high-dose group of 0.4 g/kg showed the best inhibitory impact, with an AST inhibition rate of 28% and an ALT inhibition rate of 36.5%. Liu et al. [130] used 0.2 g/kg PC to feed liver-damaged male C57BL/6 mice for 7 consecutive days after X-ray irradiation and found that PC treatment enhanced the relative mRNA expression of both glutathione (GSH-PX) and superoxide dismutase (SOD) and decreased the amount of ROS in the liver tissue. Furthermore, the level of H2AX expression in the group treated with PC was significantly lower compared with that in the group exposed to radiation. PC could significantly down-regulate downstream genes like hemeoxygenase-1 and up-regulated NF-E2-related factor 2 expression. In 2012, Sun et al. [160] used 50 mg/kg of paraquat, an organic pesticide, to induce acute pulmonary fibrosis in rats, which were then treated with 50 mg/kg of PC daily. The PC treatment decreased TGF-β1 expression, thereby reducing the number of cells expressing TNF-α and NF-κB. The positive cells expressing TNF-α and NF-κB decreased from 13.23% to 7.83% and from 6.69% to 5.01%, respectively.

### 4.4. Antidiabetic Activity

Diabetes mellitus is defined as a group of metabolic disorders characterized by high blood glucose levels brought on by abnormalities in insulin production, insulin action or both [161] and by alterations in fat, protein and carbohydrate metabolism. As reported by Ou et al. [162], PC treatment significantly reduced the weight of body, 24 h random blood glucose (RBG) and fasting plasma glucose (FPG) levels and eliminated the abnormal enlargement of islets observed in the KKAy mice pancreas. The C-PC antidiabetic impact on KKAy mice is most likely related to its capacity to boost the sensitivity of insulin, to regulate glycolipid metabolism and to ameliorate the insulin resistance of peripheral target tissues. Zheng et al. [163] conducted a study to investigate the potential of PC extracted from *S. platensis* in safeguarding against renal dysfunction and oxidative stress in db/db mice, which serve as a rodent model that mimics type 2 diabetes. The findings showed that PC oral administration (0.3 g/kg for 10 weeks) protected against renal mesangial expansion and albuminuria, and normalized tumor growth factor-β and fibronectin expression. In addition, PC was shown to normalize renal and urinary oxidative stress markers and NAD(P)H oxidase components expression. Therefore, it is concluded that PC oral administration could provide a novel and feasible therapeutic strategy for averting diabetic nephropathy. Recent investigations suggested that the antidiabetic capacity of PC might be due to the inhibition of α-glucosidase and α-amylase. An in silico analysis was conducted by Siti Halimatul Munawaroh et al. [164] to predict the molecular interactions between PC with α-glucosidase and α-amylase enzymes. According to molecular docking simulations, PC inhibits the enzymes by attaching to the active site and resulting in the disruption of substrate-enzyme binding. PC seems to have a significant role in facilitating the interaction within the active site cavities of both enzymes. According to Soni et al. [165], C-PE treatment was also shown to improve diabetic complications in streptozotocin-nicotinamide-induced type 2 diabetic rats by significantly decreasing oxidative stress and oxidized low-density lipoprotein-triggered atherogenesis. C-PE administration enhanced body weight, total protein content, and the bilirubin- and ferric-reducing capacities of plasma values and decreased food intake, organ weights, glucose serum concentrations, cholesterol, uric acid and thiobarbituric acid-reactive substances (TBARS). Furthermore, the levels of lipid hydroperoxide, TBARS, and conjugated diene in renal and hepatic tissues showed significant reductions, while there were increases in the levels of superoxide dismutase, glutathione peroxidase, catalase, reduced glutathione, vitamin C and vitamin E. 

### 4.5. Other Biological Activities 

Other activities including wound healing stimulation, neuroprotective activity, antiviral activity, immunomodulatory activity, hepatoprotective activity and intestinal flora modulation have been reported. According to the report of Soni et al. [166], it was observed that oral administration of C-PE demonstrated beneficial effects on biomarkers associated with hepatocellular, kidney, hepatobiliary and redox functions in rats exposed to CCl_4_-induced toxicity. The study concluded that orally administered C-PE might be degraded by proteolytic enzymes in the gastrointestinal tract into bilirubin and proteins with low molecular weight, thereby facilitating its pharmacological effects. According to a study elaborated upon by Ou et al. [167], C-PC demonstrated efficacy both in vitro and in vivo by providing protection against hepatocyte damage induced by CCl_4_. It can be explained by the fact that hepatocytes are shielded by C-PCs from CCl_4_-induced free radical damage. Through its anti-inflammatory properties, C-PC could be able to block inflammatory infiltration by inhibiting HGF and TGF-1 expression in CCl_4_-induced liver injury. Nemoto-Kawamura et al. [168] proposed that PC decreases allergic inflammation by suppressing antigen-specific IgE antibody and by improving biological defense activity against infectious illnesses by maintaining functions of the mucosal immune system. In their study, Pentón-Rol et al. [169] showed that C-PC exhibited the capacity to inhibit or mitigate the expression of experimental autoimmune encephalitis. Moreover, it triggered a response in regulatory T cells within peripheral blood mononuclear cells derived from patients with multiple sclerosis. PE has been suggested as a potential treatment for Alzheimer’s disease thanks to the inhibition of the beta-site amyloid precursor protein cleaving enzyme-1 (BACE1) [170]. Additionally, the antimicrobial effectiveness of PC was reported for the in vitro development of bacteria [171], viruses and fungi [172]. Also, PC is well known for possessing an anti-obesity impact that could be due to the inhibition of the pancreatic lipase activity [173]. Ma et al. [174] investigated the effect of C-PC extracted and purified from *S. platensis* on non-alcoholic fatty liver disease (NAFLD), and the findings indicate that C-PC could ameliorate inflammation and hepatic lipid accumulation in NAFLD mice by activating the AMPK signaling pathway of hepatocytes. According to Chakdar and Pabbi [82], PC lowered the levels of total cholesterol, serum cholesterol, triglyceride and low-density lipoproteins. Kavitha et al. [175] explored the atherogenic foam cell prevention efficiency of two peptides from PE on the RAW 264.7 cell line, and the results showed that both peptides reduced the foam cell formation, intracellular lipid accumulation, as well as the secretion of TNF-α and IL-6 in RAW 264.7 cell line, by targeting the scavenging receptors SRA1, CD36 and Map Kinase p38. In addition to the actions listed above, it was demonstrated that C-PC enhanced wound healing through an aurokinase-type plasminogen activator-dependent mechanism; however, its detailed molecular mechanism is still unknown [176].

## 5. Economic Valorization of PBPs from Microalgae

**Figure 5:**
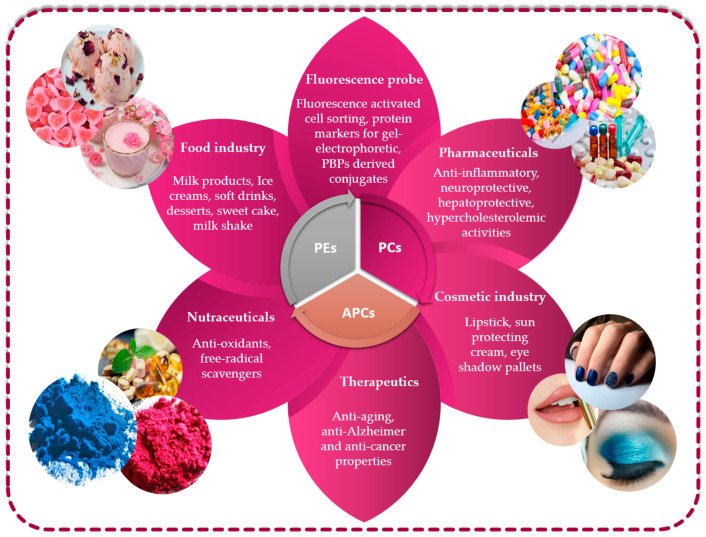
Utilizing phycobiliproteins in biotechnology: various applications and uses.

The growing market for novel bioactive substances isolated from less conventional sources justifies comprehensive searches encompassing unusual sources, as is the case of microalgae for which the PBP constituents are high-valued natural compounds with great potential for not only biotechnological pharmaceutical and nutraceutical applications but also the feed, cosmetic and food sectors (Figure 5).

### 5.1. Phycobiliproteins from Microalgae in Food Field

Currently, the human population is continuously increasing in both developed and developing countries, which is consequently leading to an increase in the global demand for food supply. Due to increasing demand and limited supply, traditional food sources are expected to face challenges in the near future in terms of both biomass production and nutritional quality. Therefore, PBPs can effectively be employed in the food industry as natural colorants. Natural dyes can help to ameliorate consumer perception in dairy products. Their use as an alternative for synthetic colorants has been extensively researched on a large scale. For example, Penna et al. [177] found that consumers positively perceived the natural dyes used in kefir, while they had a negative perception regarding artificial colors since they believed that kefir manufactured with natural colorants is healthier and tastier. Similarly, Galetovic and colleagues [178] studied the role of PBPs in the amelioration of the sensory test scores of skim milk. Moreover, these researchers determined that the ranges of temperature and pH at which this natural dye remained stable were 0 to 50 °C and 5 to 8, respectively, although at 138 °C (ultra-high temperature treatment), they still maintain their stability. In addition, the PBP extract showed great antioxidant activities with a complete absence of toxicity. García et al. [179] demonstrated also that the B-PE extracted from *P. cruentum* could be used as a coloring molecule for milk-based products. Coloring investigations were carried out to illustrate that samples containing the microalgal pink extract could be used to replicate the pink hue found in commercially available milk-based products. Moreover, the study also investigated the color stability of the three different types of dairy products during a brief period of cold storage. The findings revealed that there were no significant alterations in color observed over the 11-day analysis period, indicating that the products maintained their stability throughout this time. These findings prove the potential of the B-PE extract as a natural dye and alternative ingredient to synthetic coloring agents. In 2013, the Food and Drug Administration (FDA) endorsed PC extracted from *Spirulina* to be employed as a food dye for coloration of confections (e.g., candy and chewing gum) [180]. After that, its use in a variety of foods, including dry drinks, yogurts, ice cream, cottage cheese bread, packed cereals, blue gelatin, and other dietary and pharmaceutical supplements increased continuously [181]. *Porphyridium* genus is also utilized to produce great quantities of PE, which is used as a colorant in topping of deserts, ice cream, gelatin products and other confectionery products [182]. 

Although PBPs have several uses in the food industry, their rapid degradation in the open environment is related to their high sensitivity to light, pH and heat. The primary and most straightforward method to enhance the stability of PBPs is through the incorporation of additives. The majority of research has focused on additive utilization to increase their heat stability [183,184,185]. For instance, it has been demonstrated by Chaiklahan et al. [186] that a combination of sucrose (20%), glucose (20%) and salt (2.5%) stabilized the PC extracted from *Spirulina* sp. for long periods of time. According to Martelli and colleagues [183], the thermal stability of PBPs is significantly enhanced by a high concentration of sugar. The stabilization effect is determined by the overall concentration of added sugar rather than the specific type of sugar used. Likewise, Braga and colleagues [187] recommend the utilization of glucose to improve the heat stability of the pigment in addition to other additives like 50% sorbitol and 6% polyethylene oxide. Thus, salts (e.g., sodium chloride) and sugars (e.g., glucose) might act as protein-stabilizing agents, as they can cover the PC surface, and maintain and protect its chemical structure. In fact, the water surface tension rises when sugars are added, and consequently, the protein’s thermal stability becomes enhanced [186]. Additionally, the presence of benzoic acid, citric acid and sucrose slows the rate of thermal degradation of PE/PC [188]. PBPs have been also stabilized using other non-additive approaches such as intramolecular crosslinking between silver nanoparticles and the protein molecule [189], covalent crosslinking between α and β subunits via formaldehyde [190], complexation with PS (e.g., beet pectin and guar gum) [191] and microencapsulation techniques (e.g., extrusion, spray drying, spray cooling, centrifugal extrusion and fluidized bed drying) [192,193,194,195].

### 5.2. Phycobiliproteins from Microalgae in Biotechnology and Therapeutic Field

PBPs were revealed to be crucially important in several human activities many years ago [196]. Concisely, PBPs are employed in biomedical research as fluorescent markers and in oxidative stress-induced diseases as therapeutic agents [40,45,180,181]. They are also described as having cosmetic uses as potential non-toxic and non-carcinogenic natural dyes [180,182,183]. The numerous uses of PBPs depend on their nontoxic characteristic, as well as their biological properties, including anti-inflammatory, anticarcinogenic, antioxidative, anti-aging and protective activities [197]. Many investigations revealed that PBPs also have potential applications in memory improvement and the elimination of optics messages, quick photoelectricity detection and manual nerve networks [196,198]. Comparing PC to oral antioxidants like trolox, tocopherol and ascorbic acid, it has been discovered that PC is a more effective oxidative inhibitor in red blood cells [199,200]. According to Allan et al. [201], human breast tumor cells were diagnosed using a fluorescein isothiocyanate-conjugated anti-human leukocytic antigen antibody and a PE-conjugated anti-mouse pan-leukocyte CD45 antibody. PE is appreciated and considered as the brightest fluorophore in the world because of its intense fluorescence, suitability when excited as fluoresceins and great quantum yield. The autofluorescent property of PBPs has been utilized to develop fluorescent molecular probes for use in confocal microscopy, immunobiology, fluorescent spectroscopy, flow cytometry and single-molecule fluorescent tagging in molecular biology and experimental pharmaceutics [12]. PBPs, as opposed to synthetic fluorophores, exhibit unique spectral characteristics (e.g., large Stokes shift, low interference and quenching stability), which promote a new class of molecular tags in fluorescent applications [71,87]. It is important to note that the use of PBPs was not just limited to human diagnostics but also extended primarily to the infectious diseases of animals that are closer to humans and thereby pose health problems. In fact, PBPs were broadly employed to detect viral, as well as bacterial, pathogens of swine and poultry, including other animals like monkey, pig, mouse, rat, equines and rabbit [197]. PE has already been formulated as eyeliners and lipsticks [202]. Moreover, purple and pink cosmetics (e.g., eye shadow, face make-up and lipstick) have been prepared from various red microalgae [197]. In Japan, PC extracted from the *Arthrospira* genus has already been marketed as cosmetic dyes and formulated as an eye shadow [203]. 

## 6. Conclusions

Microalgae possess the ability to function as living factories, capable of producing great quantities of natural PBPs of industrial importance. Cultivation parameter optimization is one of the most promising strategies to produce abundant PBPs. In terms of extraction and purification processes of PBPs, a wide range of protocols and parameters have been taken into consideration; however, the ideal or optimal process still depends on the microalgal strain and on the potential application. PBPs exhibit several activities including anti-inflammatory, antidiabetic, antitumor and antioxidant activities. Thus, these PBPs play essential roles in fulfilling demands of numerous applications in the biomedical, biotechnology, pharmaceutical, natural colorants, nutraceutical, cosmetics, fluorescent and therapeutic fields. Despite the large-scale production of PBPs isolated from microalgae, there are still a number of challenges to be solved. The most common constraints in cultivation settings are their susceptibility to microbial contamination, the costly installation and operation expenses, and the demanding maintenance requirements of optimal culture conditions. Additionally, there is a lack of sufficient knowledge on microalgal photosynthetic machinery, which is one of the principal challenges related to the optimization of biosynthesis and industrial production of microalgal pigments. One of the significant limitations in the production of microalgal pigments is the rising cost associated with pigment production, particularly due to the drying of the biomass using methods like drum drying or spray drying. Until now, only a small number of microalgae have been used for the commercial production of PBPs. Therefore, extensive exploration of the diversity of microalgae is needed for PBP production. In the near future, innovative methods such as advanced gene editing technologies, along with the integration of ‘omics’ (proteomic, transcriptomic and metabolomic) analyses and computational tools, have the potential to become prominent strategies for the commercial-scale production of microalgal pigments.

## Figures and Tables

**Table 1:** Extraction procedures employed for the recovery of PBPs from microalgae.

Microalgae Species	Cell Disruption Methods	Yield/Extraction Efficiency PE	Yield/Extraction Efficiency PC	References
*Porphyridium purpureum*	Microwave-assisted extraction	73.7 ± 2.3 μg/mg	34.8 ± 6.4 μg/mg	[98]
*Rhodomonas* sp.	Sodium phosphate buffer (0.1 M, pH = 6.0) + repetitive freeze–thaw cycles + sonication for 10 min	5.36 ± 0.68%	-	[33]
*Porphyridium cruentum*	Acetate buffer (50 mM, pH = 5.5) + five repeated freeze/thawing cycles	0.27 mg/mL	-	[99]
*Porphyridium cruentum*	Freeze/thawing cycles (−20° C and 20–25° C)	71 ± 4%	-	[100]
*Porphyridium cruentum*	Freeze–thawing cycles + ultrasound	69 ± 3%	-	[100]
*Arthrospira platensis*	Freeze–thawing cycles + ultrasound	-	76 ± 6%	[100]
*Pseudanabaena amphigranulata*	Three cycles of repeated freezing in liquid nitrogen + maceration mortar and pestle	10.2 ± 3.9 mg/L	86 ± 14.7 mg/L	[101]
*Pseudanabaena catenate*	Three cycles of repeated freezing in liquid nitrogen + maceration mortar and pestle	25.5 ± 5.1 mg/L	28.8 ± 2.8 mg/L	[101]
*Spirulina maxima*	Ultrasonication	0.8 mg/mL	11.3 mg/mL	[102]
*Synechococcus* 833	Incubation of sample for 2 h at 37 °C + nitrogen cavitation cycles (1500 psi, 10 min)	-	-	[103]
*Limnothrix* sp.	Distilled water + activated carbon (1% *w*/*v*) and chitosan (0.01 g/L)	-	18%	[104]
*Porphyridium cruentum*	Homogenization in 1 M acetic acid sodium acetate buffer + sonication (10 min)	32.7%	-	[105]
*Phormidium* sp. A27DM	Freeze–thaw cycles (−30 °C and 4 °C) in 1 M Tris HCl buffer	62.6%	-	[106]
*Arthrospira platensis*	Enzymatic extraction (lysozyme)	-	APC: 2.27 mg/g	[107]
*Nostoc commune*	Pulsed electric fields	-	29.66 ± 0.52 mg/g	[108]
*Porphyridium marinum*	Sodium phosphate buffer (20 mM, pH = 7.2) + freeze–thawing cycles + ultrasound	57 mg/g	-	[11]

**Table 2:** Purification procedures applied for PBP purification from microalgae.

Microalgae Species	PBPs	Purification Methods	Yield (%)	Purity	References
*Bangia atropurpurea*	PE and PC	Gel filtration with Sephadex G-200	91.3 and 68.3	4.76 and 2.80	[113]
*Rhodella violace*	APC	Gradient centrifugation Hydroxylapatite chromatography Preparative PAGE (native)	-	-	[114]
*Synechochoccus* sp.	PC	Hydrophobic interaction chromatography Ion exchange chromatography	-	4.85	[115]
*Galdieria sulphuraria*	PC	(NH_4_)_2_SO_4_ fractionation Aqueous two-phase extraction Anion exchange chromatography	39	4.5	[116]
*Spirulina platensis*	PC	Chitosan adsorption Two-phase aqueous extraction	66	5.1	[117]
*Spirulina platensis*	PC	Chitosan adsorption Two-phase aqueous extraction Ion exchange chromatography	-	6.69	[117]
*Spirulina platensis*	PC	Expanded bed adsorption chromatography Ion exchange chromatography	8.7	3.64	[118]
*Spirulina platensis*	PC	Repeated two-phase aqueous extraction Ultrafiltration	85.0	4.05	[119]
*Pseudanabaena* sp.	PE	Precipitation with (NH_4_)_2_SO_4_ Gel filtration chromatography Ion exchange chromatography	47.0	6.86	[120]
*Nostoc* sp.	PC	Ion exchange chromatography Two-phase aqueous extraction	-	3.55	[37]
*Lyngbya* sp. A09DM	PE, PC and APC	Triton X-100 mediated with (NH_4_)_2_SO_4_ precipitation Ion exchange chromatography Gel filtration chromatography	76.16, 60.23 and 71.91	6.75, 5.53 and 5.43	[121]
*Nostoc* sp.	PE	Ion exchange chromatography Two-phase aqueous extraction	-	-	[37]
*Porphyridium marinum*	PE	Two steps of precipitation with (NH_4_)_2_SO_4_ Dialysis DEAE-cellulose exchange chromatography	72	5	[11]

## Data Availability

All raw data are readily available upon request.
